# Therapeutic effects of the euglenoid ichthyotoxin, euglenophycin, in colon cancer

**DOI:** 10.18632/oncotarget.22238

**Published:** 2017-11-01

**Authors:** April B. Cabang, Keya De Mukhopadhyay, Sarah Meyers, Jay Morris, Paul V. Zimba, Michael J. Wargovich

**Affiliations:** ^1^ Department of Molecular Medicine, University of Texas Health Science Center at San Antonio, San Antonio, TX 78229, USA; ^2^ College of Charleston, Charleston, SC 29424, USA; ^3^ Center for Coastal Studies and Department of Life Sciences, Texas A&M – Corpus Christi, Corpus Christi, TX 78412, USA

**Keywords:** euglenophycin, colon cancer, ichthyotoxin, autophagy, therapy

## Abstract

Colorectal cancer (CRC) remains one of the most commonly diagnosed cancers and the 3^rd^ leading cause of cancer-related mortality. The emergence of drug resistance poses a major challenge in CRC care or treatment. This can be addressed by determining cancer mechanisms, discovery of druggable targets, and development of new drugs. In search for novel agents, aquatic microorganisms offer a vastly untapped pharmacological source that can be developed for cancer therapeutics. In this study, we characterized the anti-colorectal cancer potential of euglenophycin, a microalgal toxin from *Euglena sanguinea*. The toxin (49.1-114.6 μM) demonstrated cytotoxic, anti-proliferative, anti-clonogenic, and anti-migration effects against HCT116, HT29, and SW620 CRC cells. We identified G1 cell cycle arrest and cell type - dependent modulation of autophagy as mechanisms of growth inhibition. We validated euglenophycin’s anti-tumorigenic activity *in vivo* using CRL:Nu(NCr)Foxn1^nu^ athymic nude mouse CRC xenograft models. Intraperitoneal toxin administration (100 mg/kg; 5 days) decreased HCT116 and HT29 xenograft tumor volumes (n=10 each). Tumor inhibition was associated with reduced expression of autophagy negative regulator mechanistic target of rapamycin (mTOR) and decreased trend of serum pro-inflammatory cytokines. Together, these results provide compelling evidence that euglenophycin can be a promising anti-colorectal cancer agent targeting multiple cancer-promoting processes. Furthermore, this study supports expanding natural products drug discovery to freshwater niches as prospective sources of anti-cancer compounds.

## INTRODUCTION

Globally, colorectal cancer (CRC) remains the 3^rd^ leading cause of cancer-related deaths [1]. The complex, heterogeneous, and continually evolving nature of cancer is a primary impediment for developing cures. Drug resistance and non-responsiveness to standard chemotherapeutics pose major challenges in cancer treatment [2]. These drive the quest for novel drugs and drug targets. Historically, terrestrial plants served as prime sources of natural compounds for drug discovery and development [3]. However, aquatic systems with a rich biodiversity potentially rivaling that of terrestrial ecosystems provide an enormously underexplored source of anti-cancer agents [4]. Compounds from marine organisms (e.g. trabectedin, cytarabine, and eribulin mesylate) have been approved for clinical trials and cancer treatment [5–7]. A few studies have reported anti-cancer molecules from freshwater sources. These include trichormamides, otteliones, and glyceroglycolipids from cyanobacteria *Trichormus* sp., plant *Otellia alismoides*, and green algae *Chlorella vulgaris* respectively, that are cytotoxic and anti-proliferative against various colon, breast, and lung cancer cell lines [8–12]. Although freshwater algae are better studied than marine algae, drug discovery efforts have been focused on marine species.

**Figure 1:**
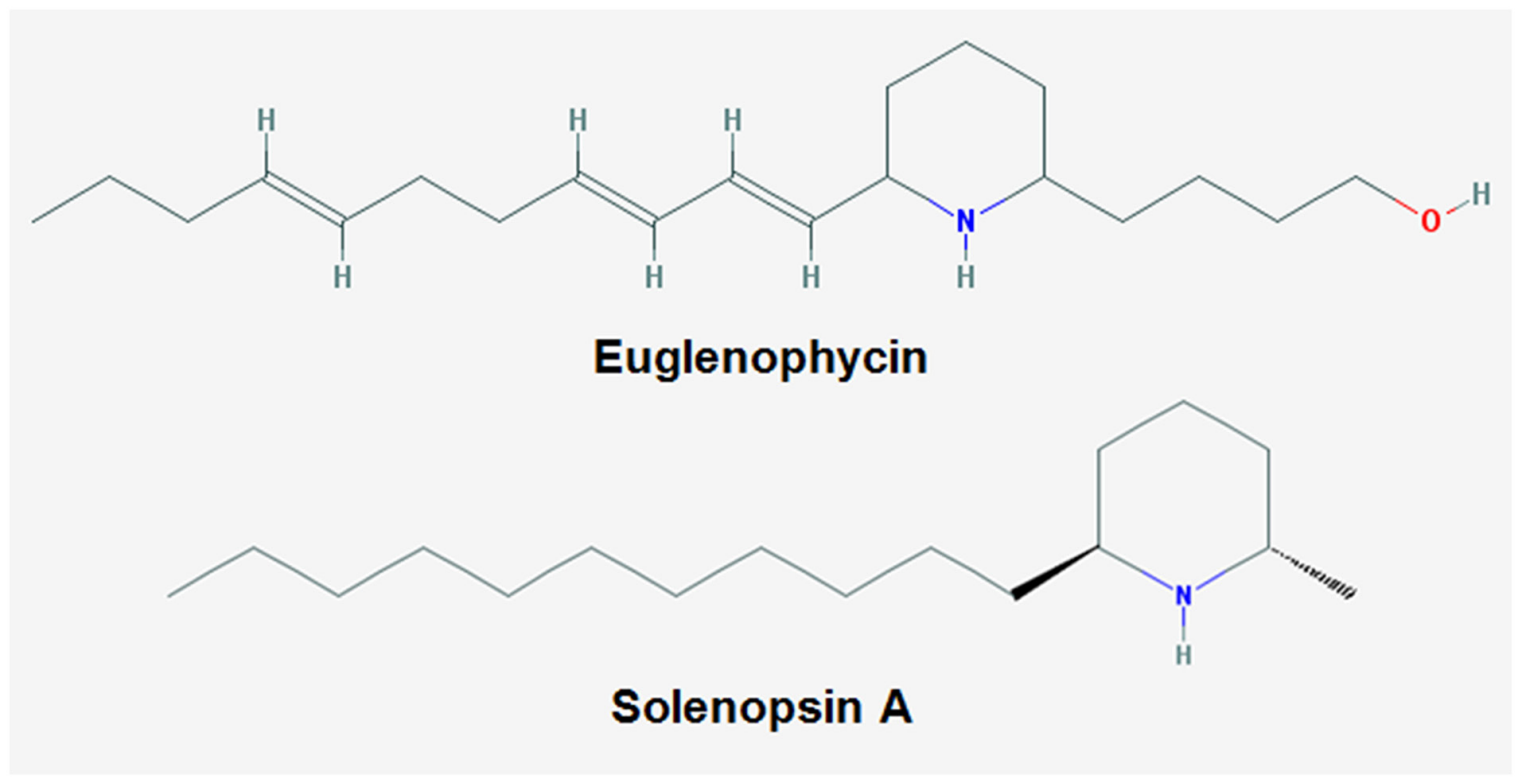
Chemical structures of euglenophycin and solenopsin A

In this study, we investigated the anti-cancer mechanisms of euglenophycin from *E. sanguinea*, a euglenoid that contributes to harmful freshwater algal blooms [13]. Overgrowth of this euglenoid has resulted in fish kills in the US and abroad, with occurrence in 4 continents [14, 15]. Previous studies by our group identified euglenophycin as a novel compound that can potentially be used for cancer treatment. Euglenophycin is structurally similar to solenopsin, an alkaloid from *Solenopsis invicta* (fire ant) venom with potent anti-cancer activity in human cancer cell lines (Figure 1) [16-19]. Euglenophycin has demonstrable cytotoxic activity against human colon and neuroblastoma cancer cell lines [13, 20, 21] that prompted us to investigate mechanisms of action in CRC cells (HCT116, HT29, and SW620) and in mouse xenograft models. We report that the anti-proliferative activity of euglenophycin is induced by G1 cell cycle arrest and autophagic modulation. The toxin also exhibits anti-migratory effects in human CRC cells and suppresses tumor growth and pro-inflammatory markers in HCT116 and HT29 xenograft models.

Autophagic dysregulation is a context-dependent (inhibition or activation) pro-survival strategy of cancer cells that is implicated in chemotherapeutic resistance [22]. Recently, aberrant autophagy has become a target for cancer treatment. Furthermore, impairing cancer-promoting inflammation and migration have been efficacious in cancer treatment and are continuously targeted for drug development [23, 24]. Euglenophycin can potentially be a good anti-cancer drug lead as it impacts proliferation, migration, autophagy, and inflammation.

## RESULTS

### Euglenophycin reduced cell proliferation and clonogenicity by promoting cell cycle arrest

**Figure 2:**
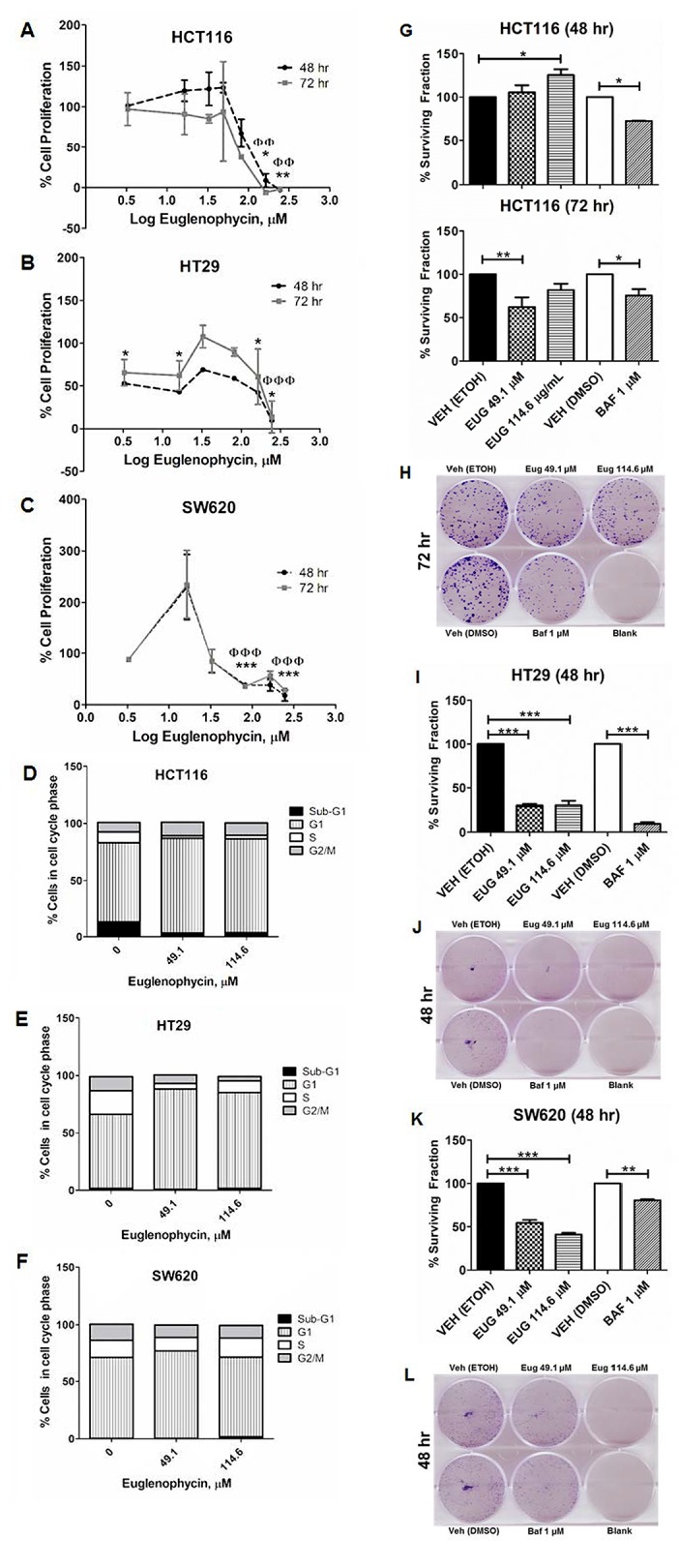
Euglenophycin inhibits proliferation and clonogenicity of HCT116, HT29, and SW620 colon cancer cell lines **(A-C)** MTT cytotoxicity assay; **(D-F)** quantified cell cycle analysis by propidium iodide staining and flow cytometry (representative images are in Supplementary Figure 1) after 48 hr toxin treatment; **(G-L)** colony formation assay visualized by crystal violet with quantified values and representative images: (G) and (H), HCT116; (I) and (J), HT29; (K) and (L), SW620. Values indicate mean ± SE (from three independent experiments). Eug is euglenophycin; Baf is Bafilomycin A; Veh is vehicle control (0 μM). (A-C) ^*^p<0.05, ^**^p<0.01 at 48 hr and ɸɸp<0.01, ɸɸɸp<0 at 72 hr. (D-H) ^*^ p<0.05, ^**^p<0.01, ^***^ p<0.001.

In characterizing potential anti-cancer properties of euglenophycin, we conducted cytotoxicity, colony formation, and cell cycle assays using 3 different CRC cell lines. The IC_50_ values of euglenophycin at 48 hr and 72 hr are as follows for: HCT116 (∼84.35 and 74.63 μM), HT29 (∼53.12 and 177.38 μM), and SW620 (∼95.32 and 129.16 μM) cells (Figure 2A-2C). Based on the IC_50_ values at 48 hr, we used 49.1 μM and 114.6 μM (near IC_50_, low and slightly higher dose) for succeeding experiments. To confirm the anti-proliferative activity and determine the cell killing mechanism(s) of euglenophycin, we conducted cell cycle analysis. Toxin treatment resulted in significant G1-phase cell cycle arrest (Figure 2D-2F; Supplementary Figure 1) in HCT116 and HT29 cells at 49.1-114.6 μM, compared to minimal effect on SW620 cells.

Furthermore, we investigated the clonogenicity of cells treated with euglenophycin or 1 μM bafilomycin A (Figure 2G-2L). Both euglenophycin and bafilomycin A diminished the colony forming ability of HT29 and SW620 cells in the 48 hr treatment. However, for HCT116, the suppressive effect of euglenophycin was observed only after 72 hr at 49.1 μM.

As cell cycle cessation is often coupled with apoptosis, we measured the degree of apoptosis using the BrdUTP-TUNEL assay counterstained with propidium iodide. Euglenophycin treatment at 49.1-114.6 μM for 48-72 hr had no significant impact on programmed cell death in all three CRC cell lines, prompting our investigation whether euglenophycin-induced cell cycle arrest is associated with autophagic activation.

### Cell type determined euglenophycin-induced autophagic activation or inhibition

**Figure 3:**
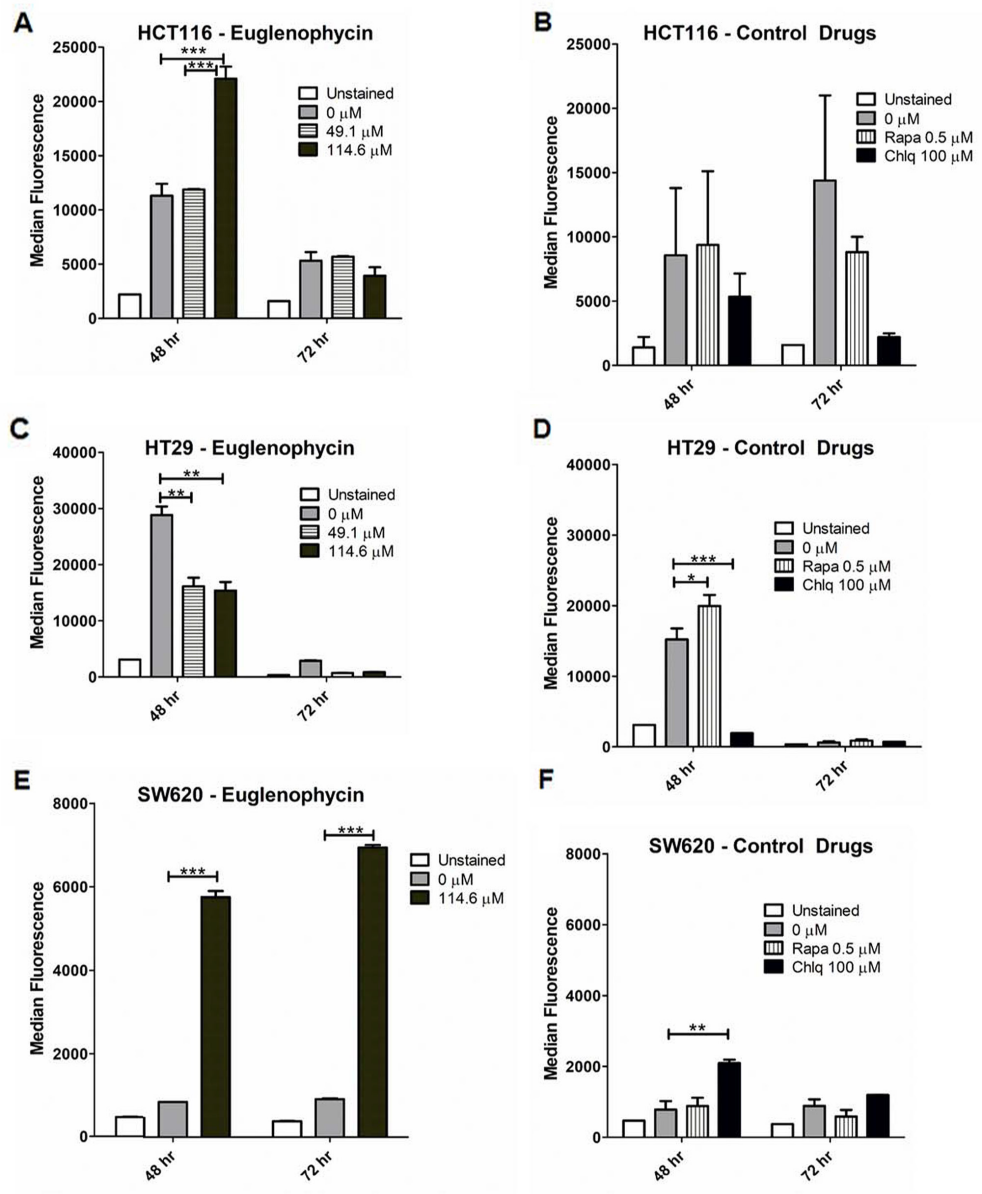
Autophagic modulatory effects of euglenophycin are cell type-dependent Alterations in autophagic flux (detected by CYTO-ID and flow cytometry) induced by treatment with euglenophycin or control drugs (autophagy activator Rapamycin or Rapa; inhibitor Chloroquine or Chlq) in **(A)** and **(B)**, HCT116; **(C)** and **(D)**, HT29; and **(E)** and **(F)**, SW620 cancer cells. Values indicate mean ± SE; ^*^ p<0.05, ^**^ p<0.01, ^***^ p<0.001.

We measured autophagic flux in all cell lines by flow cytometry (Figure 3; Supplementary Figure 2). Euglenophycin decreased autophagy in HCT116 (114.6 μM, 72 hr) and HT29 (49.1-114.6 μM, 48-72 hr) cells (Figure 3A, 3B). Differently, euglenophycin increased autophagy in SW620 (114.6 μM, 48-72 hr) and HCT116 (114.6 μM, 48 hr) cells (Figure 3A, 3E). In HCT116 cells, euglenophycin showed dose- and time-dependent alteration in the autophagic process. The autophagy activator rapamycin and inhibitor chloroquine also exhibited differential cell type, dose, and treatment time effects (Figure 3B, 3D, and 3F). Rapamycin induced autophagy in HT29 and SW620 cells at 48 hr. Chloroquine reduced autophagy in HCT116 (48-72 hr) and HT29 (48 hr) cells. Due to limited euglenophycin amounts, we were only able to investigate transcriptional levels of autophagy markers in phagophores (*Becn1 or Beclin-1*, *Atg12*, *Atg5, Lc3a, Lc3b*) and in autophagosomes/autolysosomes (*Lc3a*, *Lc3b*). As autophagic flux is translationally and post-translationally regulated, transcript levels of the autophagy markers incompletely corroborated with functional flow-cytometry results (Supplementary Figure 3). Decreased autophagy in HCT116 cells (Supplementary Figure 3A) at 72 hr was associated with reduced *Atg12* expression. However, autophagy induction in HCT116 cells at 48 hr was linked to increased *Lc3b*. In HT29 cells (Supplementary Figure 3B), autophagic inhibition was associated with reduced *Atg5* and *Atg12* expression (49.1 μM, 48 hr); *Lc3b* and *Atg12* (114.6 μM, 48 hr); *Atg5* (49.1 μM, 72 hr). In SW620 cells (Supplementary Figure 3C), autophagic activation was associated with increased *Lc3a*, *Atg5*, and *Atg12* (49.1 μM, 72 hr). To determine specific effects of autophagy activation or inhibition on gene expression levels, we treated cells with rapamycin (activator) or bafilomycin A (inhibitor) (Supplementary Figure 4). Similar to what we observed with euglenophycin treatments, gene expression of autophagy markers upon rapamycin treatment partially confirmed the functional data. Rapamycin-induced autophagy in SW620 cells (48 hr) was associated with increased levels of *Beclin-1* and *Lc3a*.

### Euglenophycin attenuated migration of HCT116 and HT29 cells

**Figure 4:**
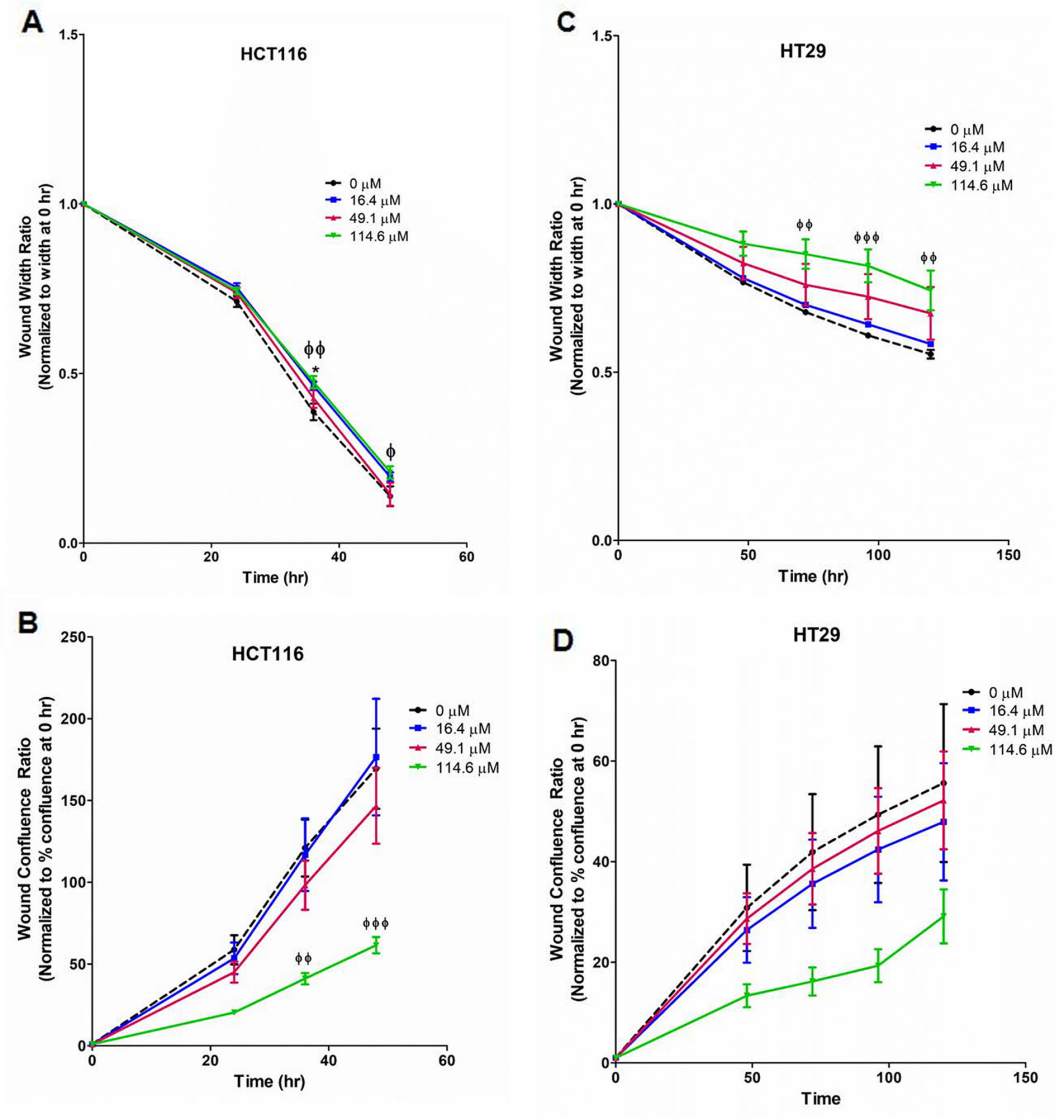
Suppression of migratory potential by euglenophycin (EUG) is cell type-dependent Wound healing measured by two parameters, wound width and wound confluence. Scratch assay quantification with **(A)** and **(B)**, HCT 116; **(C)** and **(D)**, HT 29. Representative images are in Supplementary Figure 3. ^*^p ≤ 0.05 EUG 0 μM vs 16.4 μM; ^ɸ^p ≤ 0.05 EUG 0 μM vs 114.6 μM; ^ɸɸ^p ≤ 0.01 EUG 0 μM vs 114.6 μM; ^ɸɸɸ^ p ≤ 0.001 EUG 0 μM vs 114.6 μM; values indicate mean ± SE; n=8 per treatment group.

Scratch assays were performed with HCT116, HT29, and SW620 cells. In HCT116 (Figure 4A, 4B; Supplementary Figure 5A), 114.6 μM strongly reduced cell migration at 36 – 48 hr as measured by normalized wound width and confluence. Similarly, euglenophycin treatment inhibited wound closure in HT29 cells at 72-120 hr (Figure 4C, 4D; Supplementary Figure 5B). In contrast, migration of SW620 cells was only modestly reduced by 114.6 μM toxin at 96 hr (Supplementary Figure 6).

### Euglenophycin and CPT-11 inhibited tumor growth and impacted autophagy markers in CRC xenograft models

**Figure 5:**
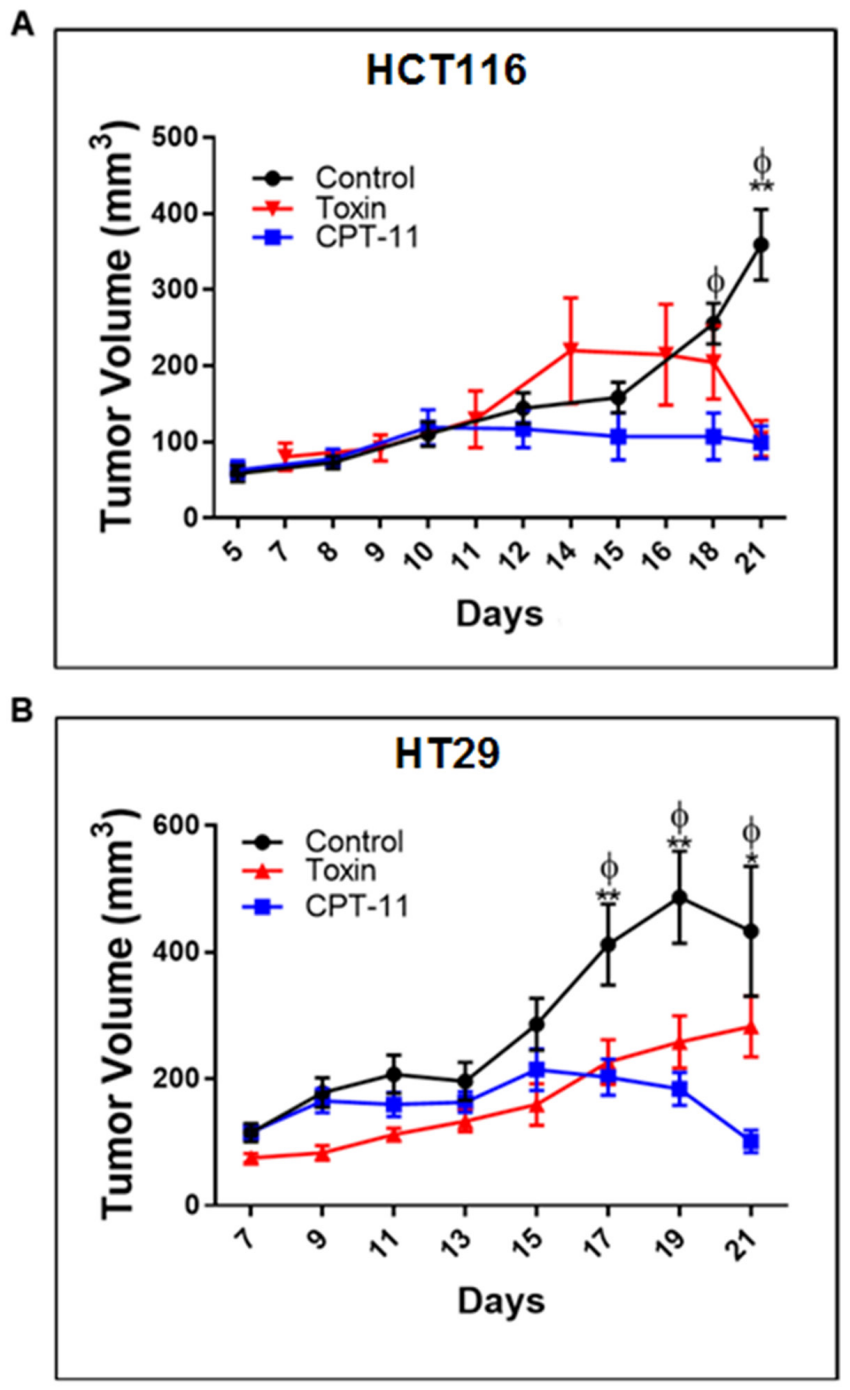
Euglenophycin inhibits *in vivo* tumor growth of colon cancer cells similarly as CPT-11 (standard chemotherapeutic) in mouse xenograft models Tumor volumes (n=10 per cell line) of mice injected with **(A)** HCT116 and **(B)** HT29 cells, respectively. ^*^p ≤ 0.05 control vs toxin (EUG); ^**^p ≤ 0.001 control vs toxin; ^ɸ^p ≤ 0.001 control vs CPT-11; values indicate ± SD. Intraperitoneal administration of CPT-11 or euglenophycin were done daily from day 10 to day 15 post-implantation.

**Figure 6:**
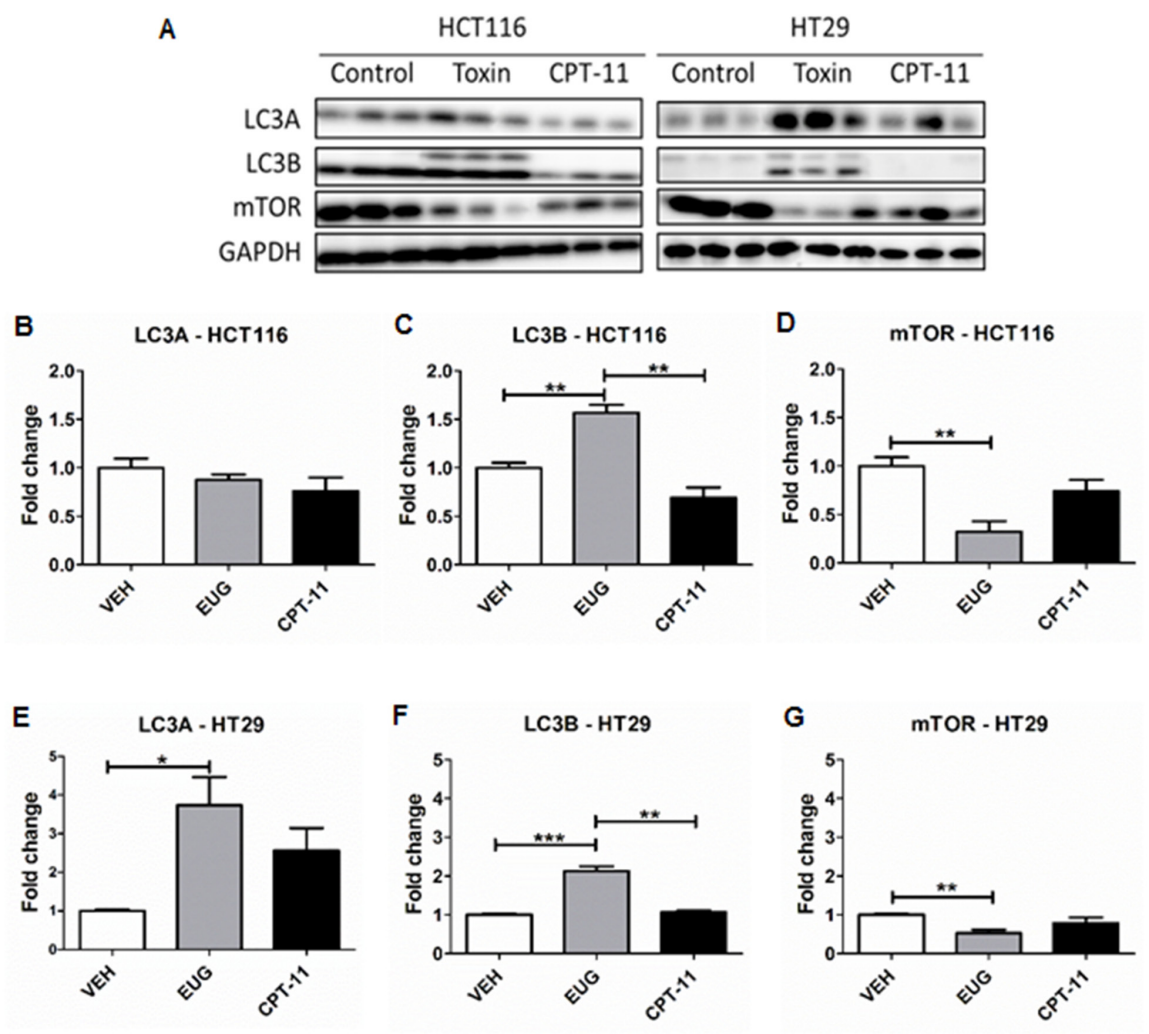
Euglenophycin (EUG) modulated protein expression of autophagy markers in HCT116 and HT29 xenografts Representative western blot is presented in **(A)** and quantification is shown in **(B-G)**. ^*^ p ≤ 0.05; ^**^ p ≤ 0.01; ^***^ p ≤ 0.001; values indicate mean ± SE (analyzed 3 tumors per group for each cell type).

To validate euglenophycin’s tumor-inhibitory activity, we used HCT116, HT29, and SW620 xenograft mouse models. Irinotecan (CPT-11) served as positive standard therapy drug. At termination, euglenophycin and CPT-11 remarkably reduced HCT116 tumor volumes compared to untreated groups (Figure 5A). Similarly, euglenophycin and CPT-11 inhibited HT29 tumor growth compared to controls starting at day 17 post-injection (Figure 5B). However, only CPT-11 inhibited tumor growth in SW620 xenografts (Supplementary Figure 7A). Tumor analyses (Figure 6) corroborated with *in vitro* data as autophagic induction indicated by increased LC3B and diminished mTOR (negative regulator of autophagy) levels were observed in euglenophycin-treated HCT116 xenografts. In contrast to *in vitro* findings, autophagic activation was observed in euglenophycin-treated HT29 xenografts indicated by increased LC3B and decreased mTOR expression. Additionally, as predicted from *in vitro* results, SW620 xenografts treated with either euglenophycin or CPT-11 had elevated autophagy demonstrated by increased LC3B and reduced mTOR expression (Supplementary Figure 7B-7E), although euglenophycin alone did not inhibit tumor growth.

### Modulatory effects of euglenophycin on serum cytokine(s) are cell-type dependent

In xenograft studies, euglenophycin or CPT-11 treatments resulted in differentially decreasing trend of pro-inflammatory cytokines/chemokines depending on the cell line (Supplementary Figure 8). Euglenophycin decreased pro-inflammatory IL12p40, IL12p70, IL1α, IL1β, IL3, IL4, IL5, IL6 and immune cells chemo-attractant proteins MCP1, MIP1α, MIP1β, and eotaxin exclusively in sera from HCT116 xenografts. Furthermore, euglenophycin reduced other inflammatory drivers TNFα, IL17, IL13, and IL2 in both HCT116 and HT29 xenografts. In addition, pro-inflammatory neutrophil chemo-attractant KC and granulocyte growth factor G-CSF were decreased in HCT116 and SW620 xenografts.

## DISCUSSION

Resistance to standard therapies is a major hurdle in treating CRC and reducing CRC-related mortality. To address this, understanding mechanisms of drug resistance, discovery of new targets, and development of drugs with well-characterized mechanisms are critical. Cancer cells that are capable of evading death machineries (apoptosis, autophagy, necrosis) give rise to drug-resistant populations. As patients develop resistance to single-molecule or pathway targeting drugs, the role of multi-targeted therapy emerges [25–27]. Therefore, a promising approach for more effective CRC treatment could involve combinations of natural products. This can be achieved by using either a cocktail of drugs acting synergistically via inhibition of multiple cancer-promoting processes or through single agents targeting a wide network of carcinogenic drivers/enablers. In this study, we characterized the anti-cancer potential of euglenophycin using CRC cell lines and mouse xenograft models. The prospective translation of this compound for multi-targeted therapy is demonstrated by its ability to impact multiple carcinogenesis pathways: aberrant proliferation, cell migration, autophagy, and inflammation. Additionally, previous study by our group demonstrated euglenophycin’s combinatorial activity with other microbial metabolites against neuroblastoma and breast cancer cell lines [20].

As cancer results from hyperproliferation with inhibition of cell death, re-establishing homeostasis is critical for both chemoprevention and oncotherapy [28]. Euglenophycin showed selective cytotoxicity against three CRC cells differing in aggressiveness (HT29 < HCT116 < SW620), with minimal effect on normal intestinal epithelial IEC-6 cells (Supplementary Figure 9). Euglenophycin promoted cell cycle arrest in all cell lines, without significantly affecting the percentage of cells undergoing apoptosis. As apoptosis and autophagy are two modes of non-necrotic death – [29], we investigated euglenophycin’s impact on macro-autophagy. Additionally, the structural resemblance of euglenophycin to solenopsin A, which exhibits similar activity to the apoptosis/autophagy regulator ceramide [16], provided a rationale to investigate euglenophycin’s effect on autophagy. Autophagy functions as both stress response and waste disposal. In oncogenesis, cancer cells either downregulate or upregulate this pathway to promote survival and escape immune surveillance [30, 31]. Several autophagy activators and inhibitors are being studied for therapeutic applications. In some cancers, inhibitory effects of autophagy activators have been reported (e.g. tamoxifen in triple negative breast cancer, fasudil + clioquinol in glioblastoma, and salinomycin in hepatocellular carcinoma) [32–34]. Paradoxically, autophagy has dual roles depending on disease stage. At an early stage, decreased autophagic flux may promote tumor initiation by elevated ROS and genomic instability. In advanced stages, increased autophagy fuels cancer cell survival by supplying cells with nutrients [35]. Combinatorial studies of autophagy inhibitors and chemotherapy enhanced therapeutic effects (e.g. 3-methyladenine or bafilomycin A and temozolomide in malignant glioma cells, 3-methyadenine and 5-fluorouracil in CRC cells) [36, 37]. In the current study, euglenophycin inhibited autophagy in HCT116 and HT29 CRC cells, while activated it in SW620 cells. These cell type-specific effects may reflect the aggressiveness and disease state from where the cells were isolated (HCT116 and HT29 from primary tumor; SW620 from metastatic site) and p53 status of each line (HCT116 p53^+/+^, HT29 mt R273H, SW620 mt R273H + P309S) [38]. With extensive characterization, the toxin can potentially be used to inhibit early-stage colorectal cancer or increase sensitivity to therapies based on the patient’s autophagic profile (activated or downregulated).

To validate euglenophycin’s bioactivity *in vivo*, we used mouse xenograft models treated with either euglenophycin or CPT-11. As predicted from *in vitro* results, euglenophycin treatment inhibited tumor growth similarly as CPT-11 in HCT116 and HT29 xenografts. Reduction in tumor growth was associated with activation of autophagy in HCT116 and HT29 xenografts implicated by LC3B elevation which marks the conjugation of phosphatidylethanolamine to LC3A (an essential step in autophagosome formation) and inhibition of the autophagy negative regulator mTOR. Moreover, euglenophycin treatment decreased serum pro-inflammatory cytokines, suggesting that euglenophycin alters tumor inflammatory microenvironment, subsequently diminishing tumor-promoting inflammation. Interestingly, combining *in vitro* and *in vivo* HCT116 and HT29 data suggest that euglenophycin is possibly an mTOR inhibitor. Euglenophycin-treated HCT116 and HT29 tumor xenografts demonstrated reduced mTOR and elevated LC3B protein expression. *In vitro*, euglenophycin induced time and dose specific increase in transcript levels of LC3B in HCT116 and Beclin-1, Atg12, and LC3B in HT29. As mTOR inhibition leads to autophagic activation, the observed increase in markers of autophagosome initiation/formation may indicate euglenophycin’s possible antagonistic effect on mTOR. Euglenophycin’s lack of inhibitory effect on the highly aggressive SW620 xenografts indicates that metastatic cells require more rigorous treatment. Although CPT-11 inhibited SW620 tumor growth and downregulated autophagy by mTOR inhibition, apoptotic activity of the drug possibly contributed to tumor reduction. Thus, future studies using a combination of euglenophycin and chemotherapy can potentially reveal a more efficacious regimen with minimal side-effects on normal cells.

In this study, the small quantity of toxin isolated from *E. sanguinea* batch cultures was the major limitation. To scale-up euglenophycin production, chemical or biosynthetic approaches need to be developed. Subsequently, with more available toxin, it is important to assess euglenophycin’s effects on: (1) the mTOR signaling pathway, (2) autophagy modulating complexes (e.g. Beclin-1/vacuolar sorting protein 34, Atg5/Atg12) [39], (3) p53^+/+^, p53 mutant, and p53^-/-^ cells and animal models of CRC as context-dependent tumor-promoting or inhibitory functions of autophagy can be influenced by p53 status [40, 41]. This will plausibly mimic responses of CRC patients with varying p53 status. Additionally, it is imperative to expand mechanistic and animal studies by determining effects of euglenophycin on proliferative, autophagy, epithelial-mesenchymal, and inflammatory biomarkers. As autophagy is involved in immune cell regulation and inflammatory response, investigating whether euglenophycin’s anti-inflammatory effects are autophagy-dependent is valuable [42]. Moreover, evaluating euglenophycin’s impact on angiogenesis will be relevant based on structural similarity to the anti-angiogenic compound solenopsin.

In conclusion, we have shown that euglenophycin, an algae-derived natural product can be utilized as a potential anti-colorectal cancer candidate by impacting cell proliferation, autophagy, migration, and inflammation. Furthermore, it would be interesting to investigate if euglenophycin can be used as an adjuvant to potentiate the efficacy of current chemotherapeutics. To our knowledge, this is the first report demonstrating tumor-inhibitory efficacy of a euglenoid compound. Thus, our findings support the inclusion of the genus *Euglena* as natural products drug discovery sources. Consequently, this study supports extending drug discovery efforts to freshwater ecosystems, with the goal of finding prospective drug leads with unique chemical structures or modes of action.

## MATERIALS AND METHODS

### *E. sanguinea* culture and euglenophycin isolation

*E. sanguinea* Ehrenberg was grown in AF6 media as previously described [13]. Briefly, 18 L cultures were grown at 24°C under 14:10 hr Light:Dark illumination. Cells were pelleted (3000 RPM, 10 min) and frozen at -80°C. Toxin was extracted and subjected to mass-directed purification using a LUNA C18 column (3x150 mm, 3 um particle size; Phenomenex, Torrance, CA) on an Agilent HP1200 system - MS6130 mass spectrometer (Agilent, Santa Clara, CA), as previously described [13]. Toxin purity was confirmed using a HP1260 HPLC equipped with an Agilent 6130c triple quadrupole mass spectrometer.

### Cell culture

HCT116, HT29, SW620 CRC lines and IEC-6 normal rat intestinal epithelial cell line were purchased from ATCC (Manassas, VA) within six months of experiments. Cells were grown in McCoy’s 5A medium (HCT116 and HT29); Leibovitz’s L-15 medium (SW620); or Dulbecco’s modified Eagle’s medium (IEC-6) supplemented with 10% FBS and 1% Pen/Strep. For IEC-6, 0.1 U/mL bovine insulin (Sigma-Aldrich, St. Louis, MO) was added to the growth medium. Cells were incubated at 37°C and 5% relative CO_2_ (HCT116, HT29, IEC-6). All culture media were purchased from Cellgro (Manassas, VA).

### MTT assay

Cells were seeded in 96-well plates with the following densities: 7,500 cells/well HCT116, 10,000 cells/well HT29, and 15,000 cells/well SW620. Cells were cultured overnight in complete media, serum-starved for 24 hr, and treated with euglenophycin (0-245.5 μM) for 48-72 hr. Subsequently, 10 μl of 12 mM MTT (Life Technologies; Carlsbad, CA) solution was added to each well, incubated for 4 hr at 37°C, and neutralized with DMSO. Absorbance was measured at 540 nm and percent viability was calculated.

### Cell cycle analysis

Cells were plated (500,000 cells/well) in 6-well plates for 24 hr under standard conditions followed by 24 hr serum starvation. Cells were treated with euglenophycin (0-114.6 μM) for 48-72 hr. Cells were trypsinized, centrifuged at 1400 rpm (4°C, 5 min), washed twice with PBS, and fixed in 70% ethanol (1x10^6^ cells/tube). Fixed cells were washed with PBS and stained in 350 μL PBS containing 10 μg/mL propidium iodide and 1 μg/mL RNase A (Sigma-Aldrich, St. Louis, MO) for 30 min at room temperature. Data were acquired using a FACSCalibur flow cytometer (BD BioSciences, San Jose, CA) and analyzed with FlowJo version 7.6.5 software (Tree Star, Inc., Ashland, OR). Singlets were gated and doublets were discriminated in all samples.

### Clonogenic assay

HCT116, HT29, and SW620 cells were treated with 0-114.6 μM euglenophycin or 1 μM autophagy inhibitor bafilomycin A for 48-72 hr. Cells were harvested, washed with PBS, and seeded in 6-well plates: HCT116 (350 cells/well), HT29 (650 cells/well), and SW620 (1000 cells/well) in complete growth medium. Colonies were allowed to form for 7-12 days. Subsequently, plates were washed with PBS, fixed, and stained with 0.5% crystal violet solution for 15 min. Plates were rinsed and air dried. Plating efficiency (PE) and surviving fraction (SF) were calculated as follows: PE = (number of colonies formed/number of colonies seeded) x 100; SF = (PE of treatment/PE of control) x 100.

### Autophagy assay

HCT116, HT29, and SW620 cells were treated with either 0-114.6 μM euglenophycin, 0.5 μM rapamycin, or 100 μM chloroquine for 48-72 hr. Autophagy was detected using CYTO-ID autophagy detection kit (Enzo Life Sciences; Farmingdale, NY) following the manufacturer’s recommended protocol. All samples were analyzed in the FITC-channel using a BD LSR II flow cytometer (BD Biosciences, San Jose, CA). Data analysis with doublet discrimination was done using FlowJo version 7.6.5 software.

### Gene expression analysis of autophagy markers

HCT116 (300,000 cells/well), HT29 (450,000 cells/well), and SW620 (450,000 cells/well) cells were seeded in 6-well plates in complete medium for 24 hr. Subsequently, cells were treated for 48-72 hr with 0-114.6 μM euglenophycin or control drugs (0.5 μM Rapamycin; 1 μM Bafilomycin A) after 24 hr serum starvation. RNA was isolated using Aurum^™^ total RNA mini kit and cDNA synthesis (from 0.4 μg total RNA) was done with iScript cDNA synthesis kit (Bio-Rad, Hercules, CA) according to the manufacturer’s protocol. Verified qRT-PCR SYBR^®^ Green primers for *Lc3a*, *Lc3b*, *Becn1*, *Atg5*, *Atg12*, and *Gapdh* were purchased from Sigma-Aldrich (Supplementary Table 1). qRT-PCR was done using SsoAdvanced Universal SYBR Green Supermix and CFX96 Touch^™^ real time PCR detection system (Bio-Rad, Hercules, CA). The cycling parameters are as follows: initial denaturation 95°C, 2 min; denaturation 95°C, 15 s; annealing/extension 54°C, 30 s; number of cycles 40; melt curve 65°-95°C (0.5°C increments). The comparative CT (2^-ΔΔCT^) method was used for all quantification. Values were normalized to GAPDH.

### Cell migration assay

HCT116, HT29, and SW620 cells were cultured in 96-well plates in complete growth medium at the following densities (cells/well): HCT116 (12,500), HT29 (15,000), and SW620 (20,000). A monolayer scratch was performed using a WoundMaker and visualized using the IncuCyte ZOOM real time imaging system (Essen BioScience, MI, USA). Cells were treated with 0-114.6 μM euglenophycin and imaged at 3 hr intervals for 72-120 hr to monitor cell migration and wound healing.

### Tumor studies

All animal experiments were conducted following the University of Texas Health at San Antonio Institutional Animal Care and Use Committee guidelines. In total, 5 x 10^6^ HCT116, 8 x 10^6^ HT29, and 4 x 10^6^ SW620 colon cancer cells were injected subcutaneously into CRL:NU(NCr)Foxn1^nu^ athymic nude male mice (Charles River, Wilmington, MA; n=5 animals per cell line; n=10 tumors per cell line). Animals were injected with either 100 mg/kg irinotecan (CPT-11) as control drug or 100 mg/kg euglenophycin for 5 days (days 10-15 post-injection). Untreated control animals were injected with vehicle (ethanol or DMSO). Tumor volumes were measured using the formula V = (4/3) × π × (W/2)^2^ × (L/2) [43]. Animals were sacrificed at day 21.

### Protein expression analysis

Xenograft tumors were excised and proteins were isolated using T-Per Tissue buffer (Thermo Fisher Scientific; Waltham, MA) and homogenizer (IKA Ultra-Turrax, Fisher Scientific, Waltham, MA). Tissues were homogenized for 10 s followed by 10 min lysis, 10 min centrifugation at 13,000 rpm, and storage of supernatants at -80°C. Protein concentrations were measured using Pierce BCA protein assay kit (Thermo Fisher Scientific, Waltham, MA). For western blotting, 35 μg protein samples were separated by SDS-PAGE (12% acrylamide), transferred to PVDF membranes, and blocked with 5% milk in TBST buffer. Blots were incubated overnight with the following antibodies purchased from Abcam (Cambridge, MA) or Cell Signaling (Danvers, MA): LC3A (1:500; ab52628, Abcam), LC3B (1:500; ab168831, Abcam), mTOR (1:500; 2972, Cell Signaling). GAPDH (1:2500; ab9485, Abcam) was used as loading control and HRP-conjugated goat anti-rabbit (1:5000, ab6721, Abcam) as secondary antibody. For detection and imaging, Clarity Western ECL substrate and ChemiDoc touch imaging system (Bio-Rad, Hercules, CA) were utilized following manufacturer’s protocol.

### Cytokine and chemokine assay

Serum cytokine/chemokine profile drawn at termination were determined using the Bio-Plex Pro group 1 mouse cytokine 23-plex assay kit and analyzed with the Bio-Plex 200 Luminex-based multiplex analysis system (Bio-Rad, Hercules, CA). Serum from all treatment groups for HCT116, HT29, and SW620 were analyzed (n=4/group).

### Statistical analyses

Data analyses were performed by *t*-test or analysis of variance (ANOVA) followed by Tukey’s multiple comparison post-test, as appropriate. Data are presented as mean ± SE or ± SD, as indicated. Statistical analyses were done using GraphPad Prism version 6 software (GraphPad Software, Inc., La Jolla, CA).

## SUPPLEMENTARY MATERIALS FIGURES AND TABLE


