# Supplementary material for: Therapeutic effects of the euglenoid ichthyotoxin, euglenophycin, in colon cancer
Source: Oncotarget. 2017 Nov 1;8(61):104347–58. doi: 10.18632/oncotarget.22238 (PMC5732811; doi:10.18632/oncotarget.22238)
Supplement: Supplementary file 1 [file oncotarget-08-104347-s001.pdf]

Therapeutic effects of the euglenoid ichthyotoxin, euglenophycin, in colon cancer

SUPPLEMENTARY MATERIALS

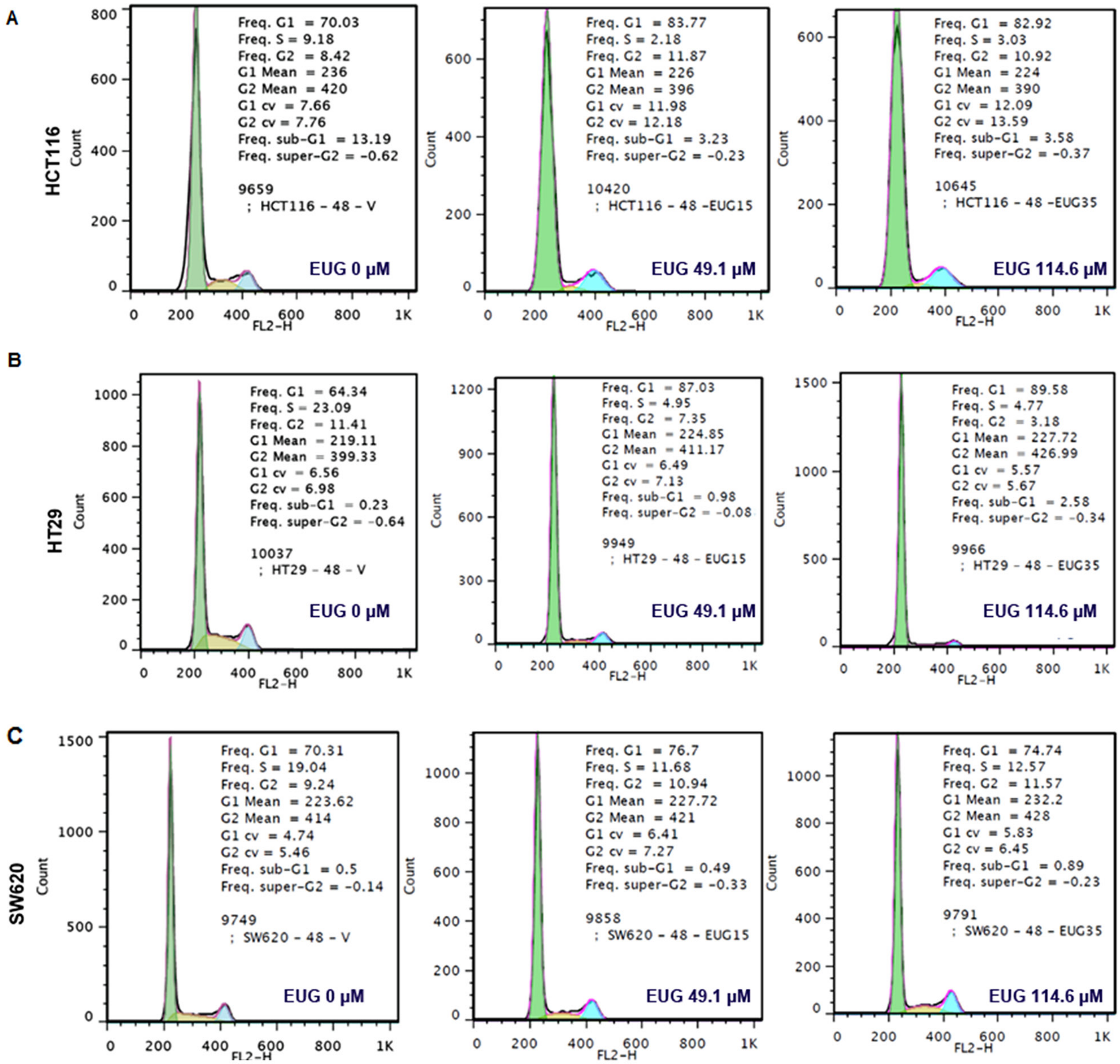

Supplementary Figure 1: Cell cycle analysis representative histograms of **(A)**, HCT116; **(B)**, HT29; **(C)**, SW620 cells treated with euglenophycin.

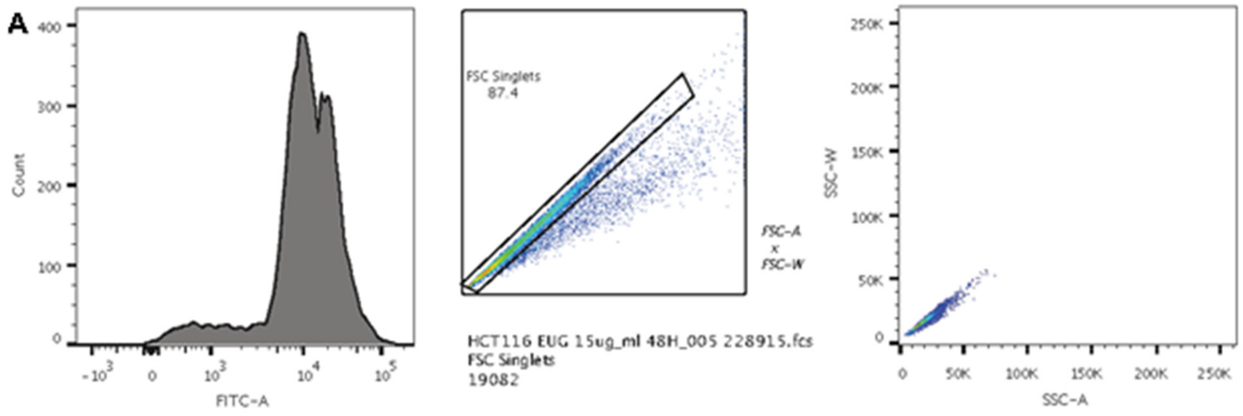

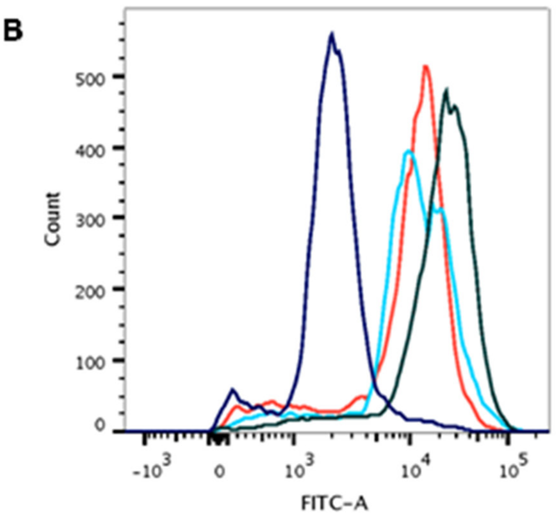

Sample Name	Subset Name	Count
HCT116 UNSTAINED 48H 228903.fcs	FSC Singlets	19109
HCT116 EUG 35ug_ml 48H_006 228917.fcs	FSC Singlets	18600
HCT116 EUG 15ug_ml 48H_005 228915.fcs	FSC Singlets	19082
HCT116 V ETOH 48H_004 228913.fcs	FSC Singlets	19139

FSC Singlets: Mean :*FSC-A*: 45258
FSC Singlets: Median :*FITC-A*: 11936
FSC Singlets: Median :*FSC-A*: 34496

Supplementary Figure 2: Detection of autophagic flux. **(A)**, Representative gating and doublet discrimination strategy in detection of autophagic flux using CYTO-ID kit and flow-cytometry; **(B)**, histogram and quantification using cell count and median fluorescence.

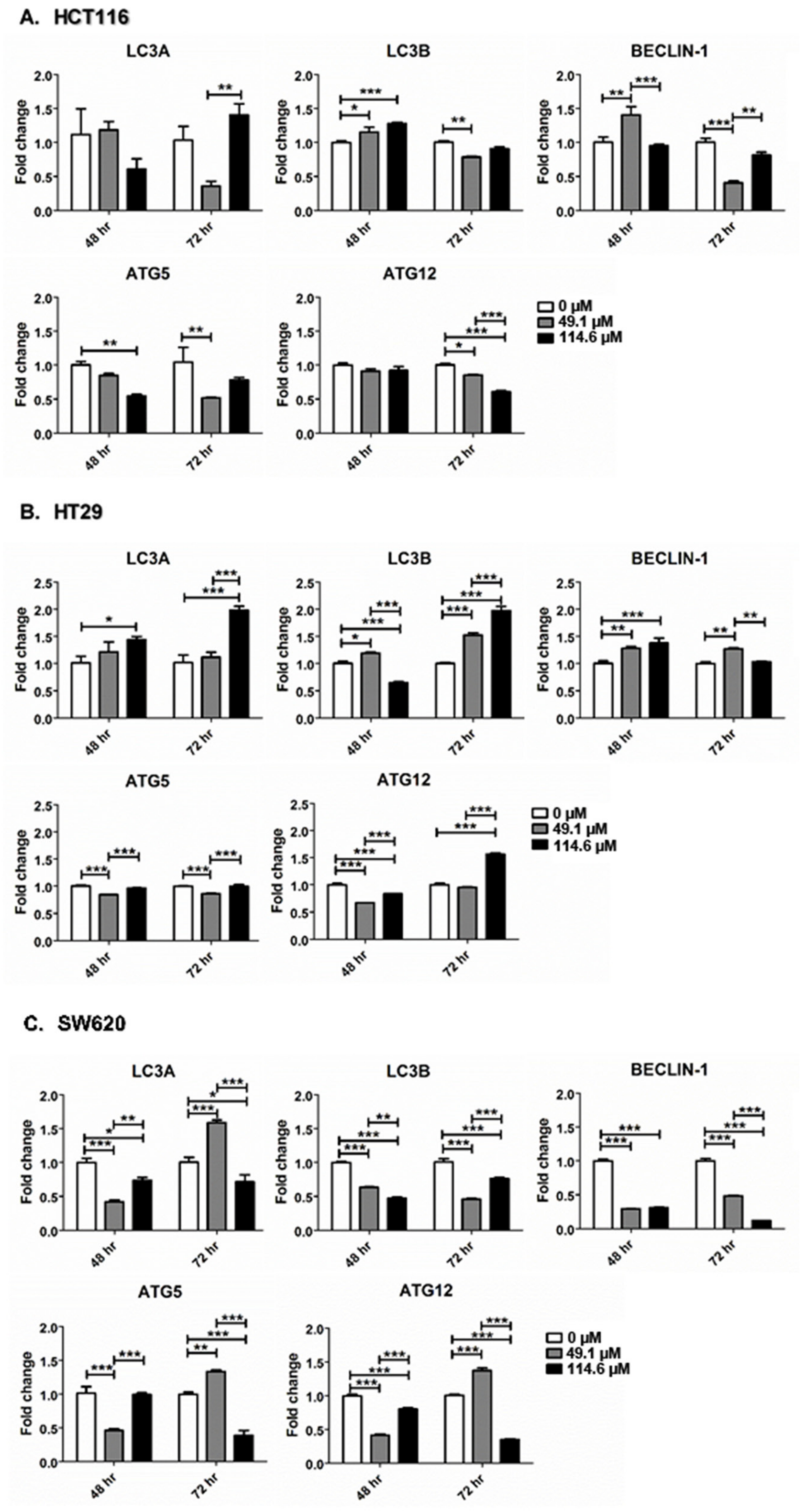

Supplementary Figure 3: qRT-PCR of autophagy markers in **(A)** HCT116; **(B)**, HT29; and **(C)**, SW620 cells treated with euglenophycin. Values indicate + SE from three independent experiments.

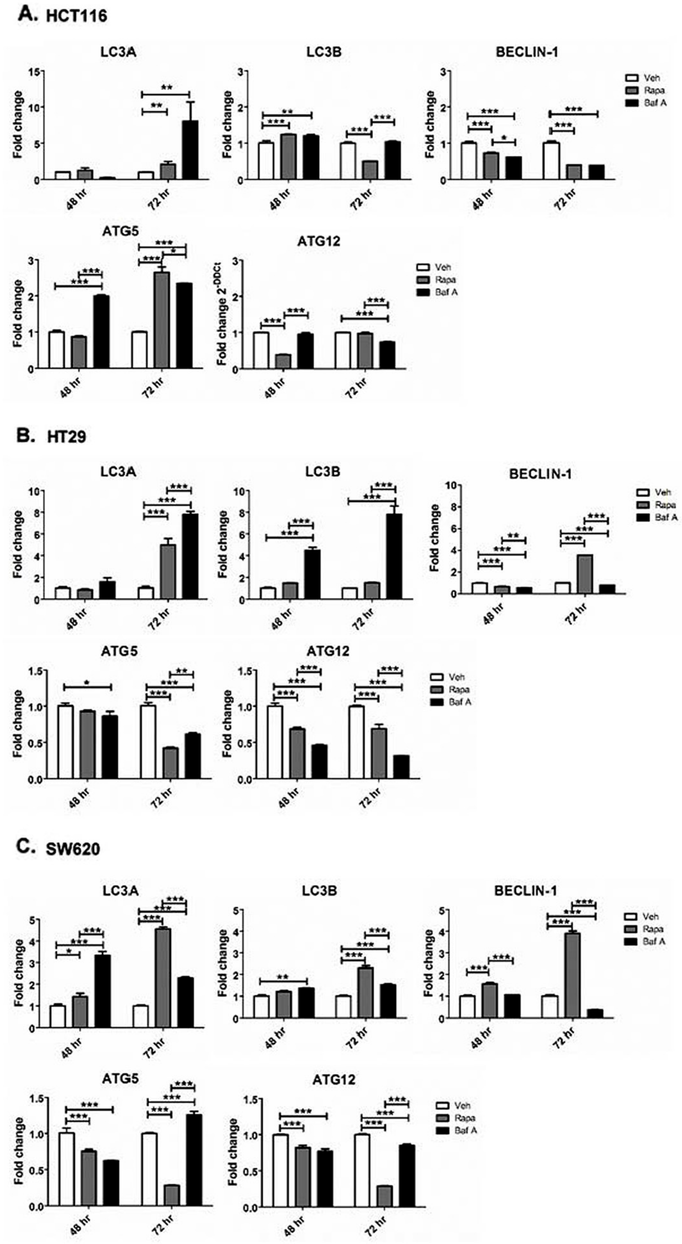

Supplementary Figure 4: qRT-PCR of autophagy markers in **(A)**, HCT116; **(B)**, HT29; and **(C)**, SW620 cells treated with rapamycin (Rapa) or Bafilomycin A (Baf A)**.** Values indicate + SE from three independent experiments.

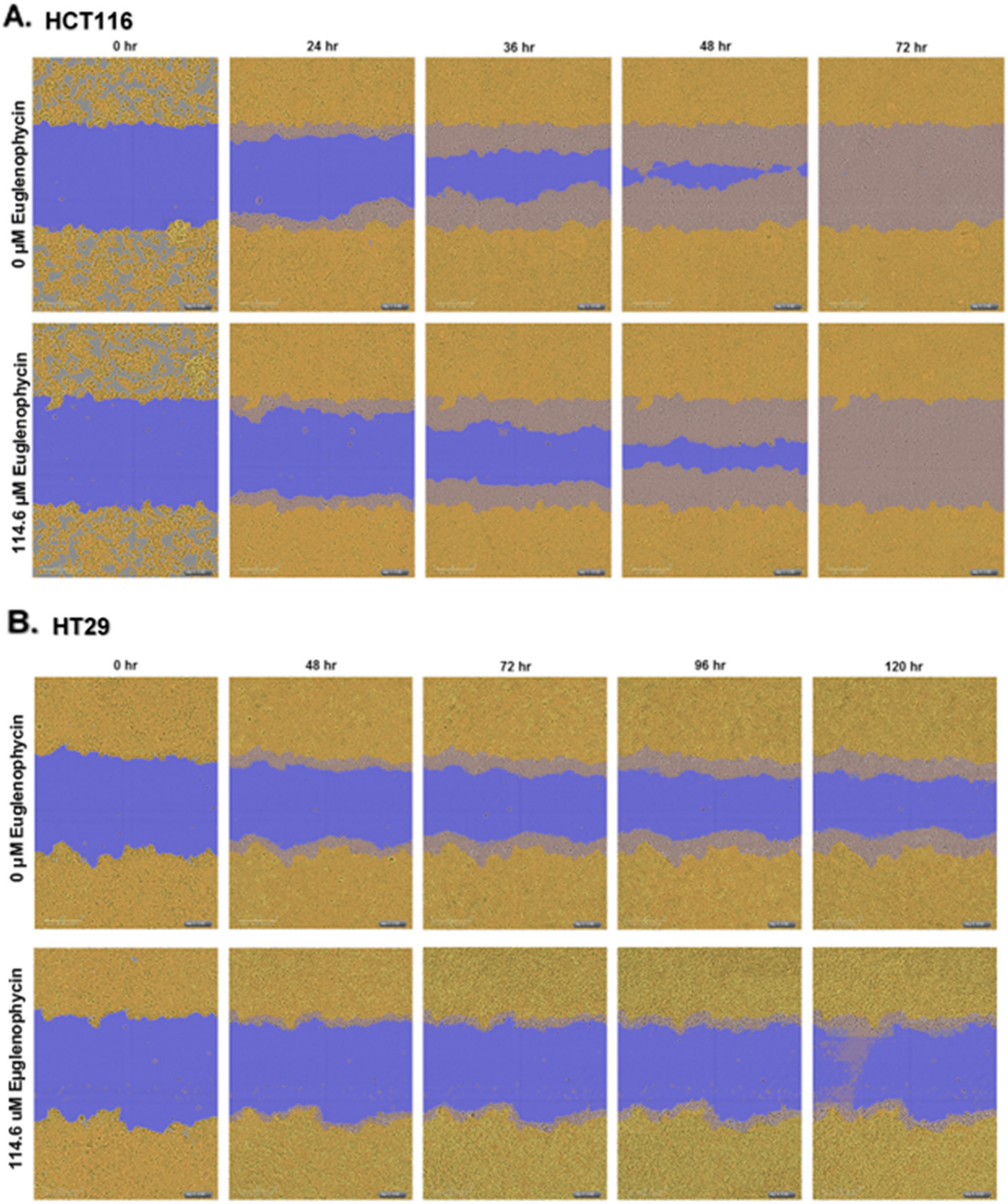

Supplementary Figure 5: Scratch assay representative images of **(A)**, HCT116 and **(B)**, HT29 cells. Regions are color coded as follows: wound area (blue), non-migrating cells (golden yellow), migrating cells that populate the wound (light brown).

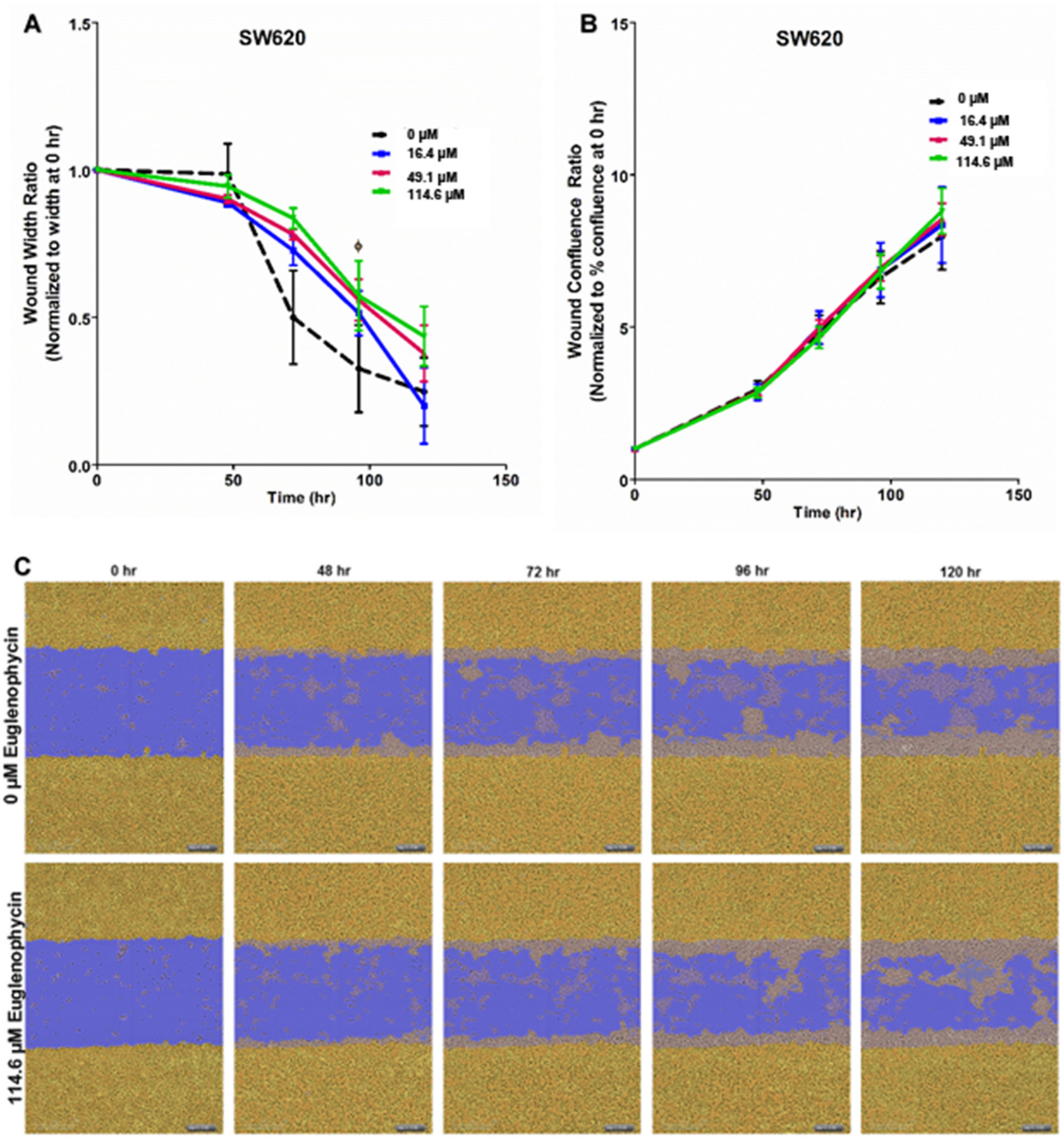

Supplementary Figure 6: Effect of euglenophycin on migration of SW620 cells. **(A-B)** Quantified wound width and confluence; **(C)** scratch assay representative images. Regions are color coded as follows: wound area (blue), non-migrating cells (golden yellow), migrating cells that populate the wound (light brown).

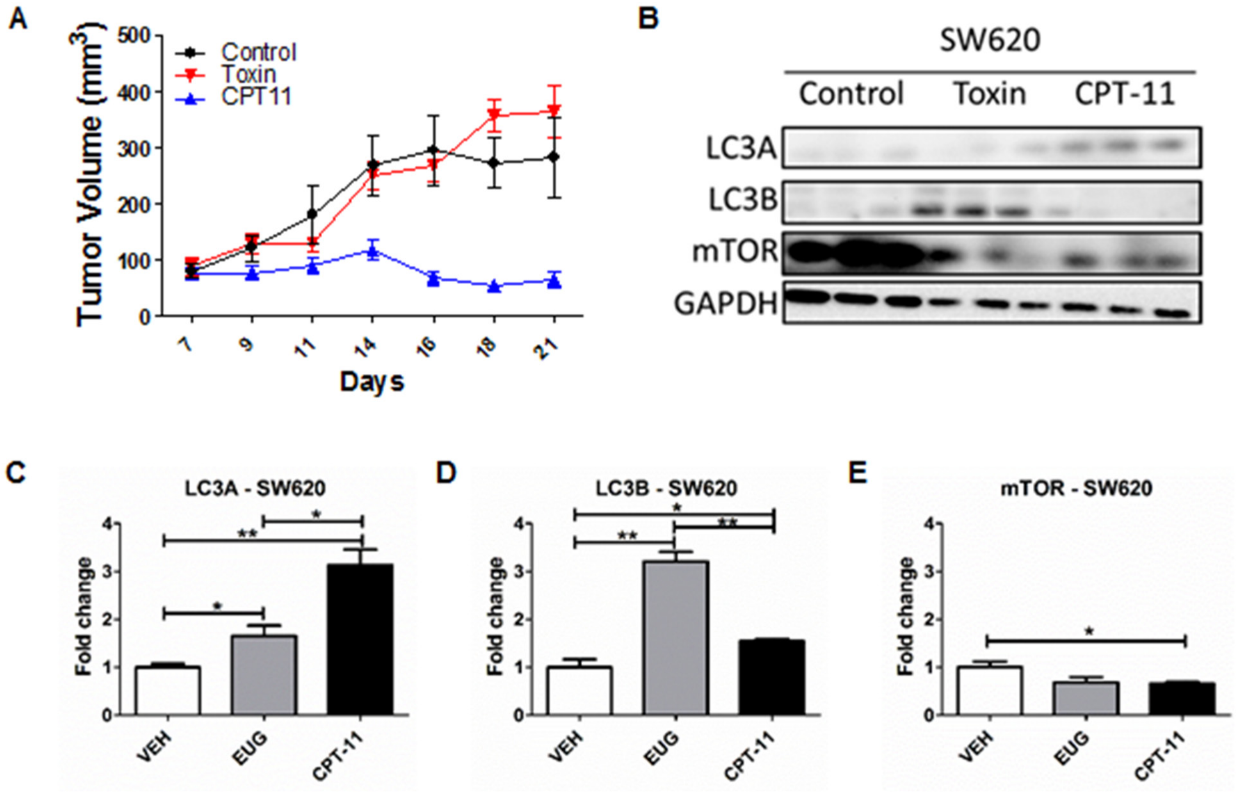

Supplementary Figure 7: Measurement of tumor growth and autophagy markers in SW620 mouse colorectal cancer xenografts treated with either euglenophycin or CPT-11. **(A)** Tumor measurements (values indicate mean ± SD) and protein expression of autophagy markers in **(B)**, representative western blot image and quantification in **(C-E)** (values indicate mean ± SE). $^{*}p \leq 0.05$ and $^{**}p \leq 0.001$.

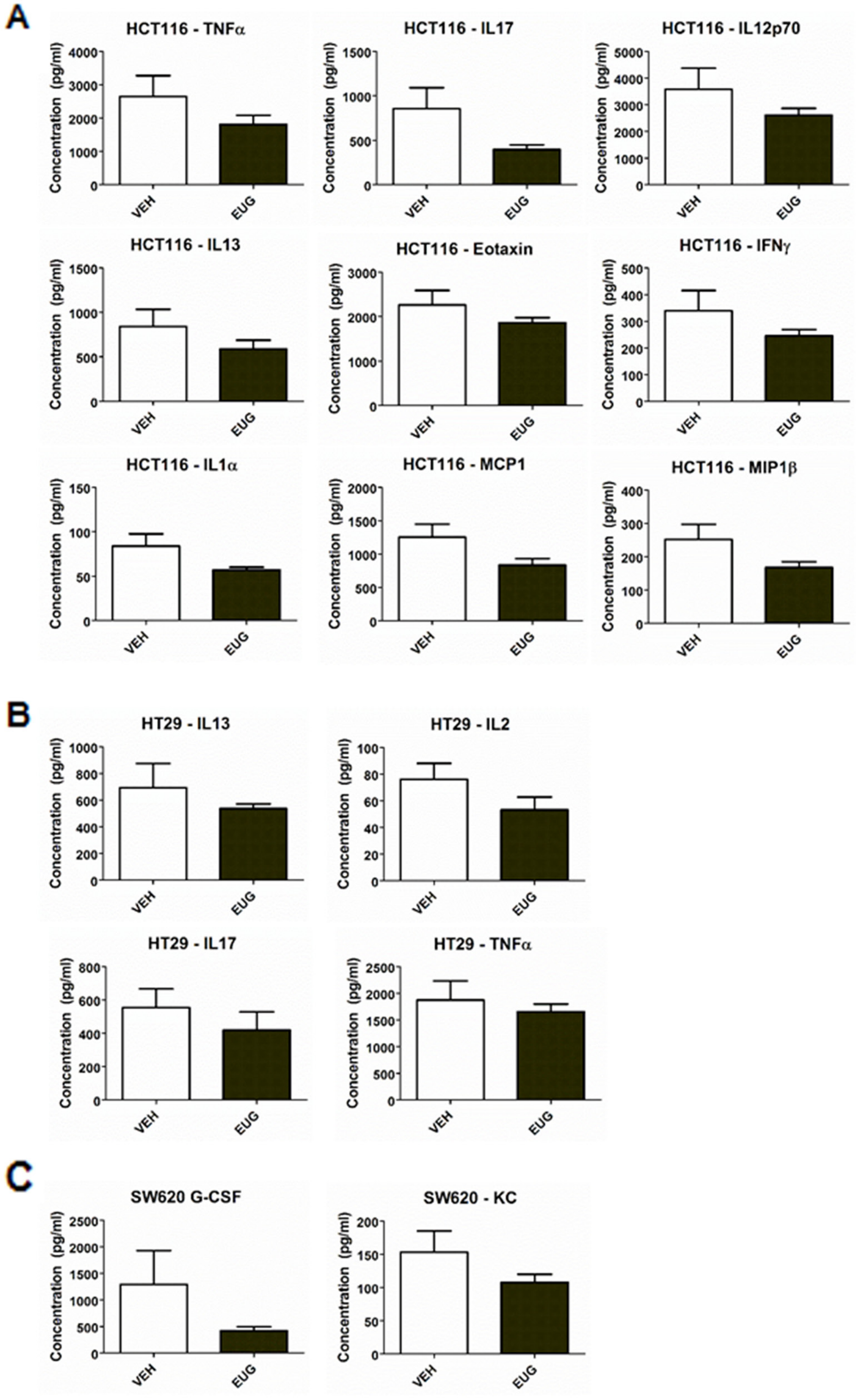

Supplementary Figure 8: Euglenophycin differentially affected *in vivo* serum levels of multiple cytokines/chemokines depending on cell type. Representative graphs of several cytokines/chemokines decreased in **(A)** HCT116; **(B)** HT29, and **(C)** SW620 xenografts.

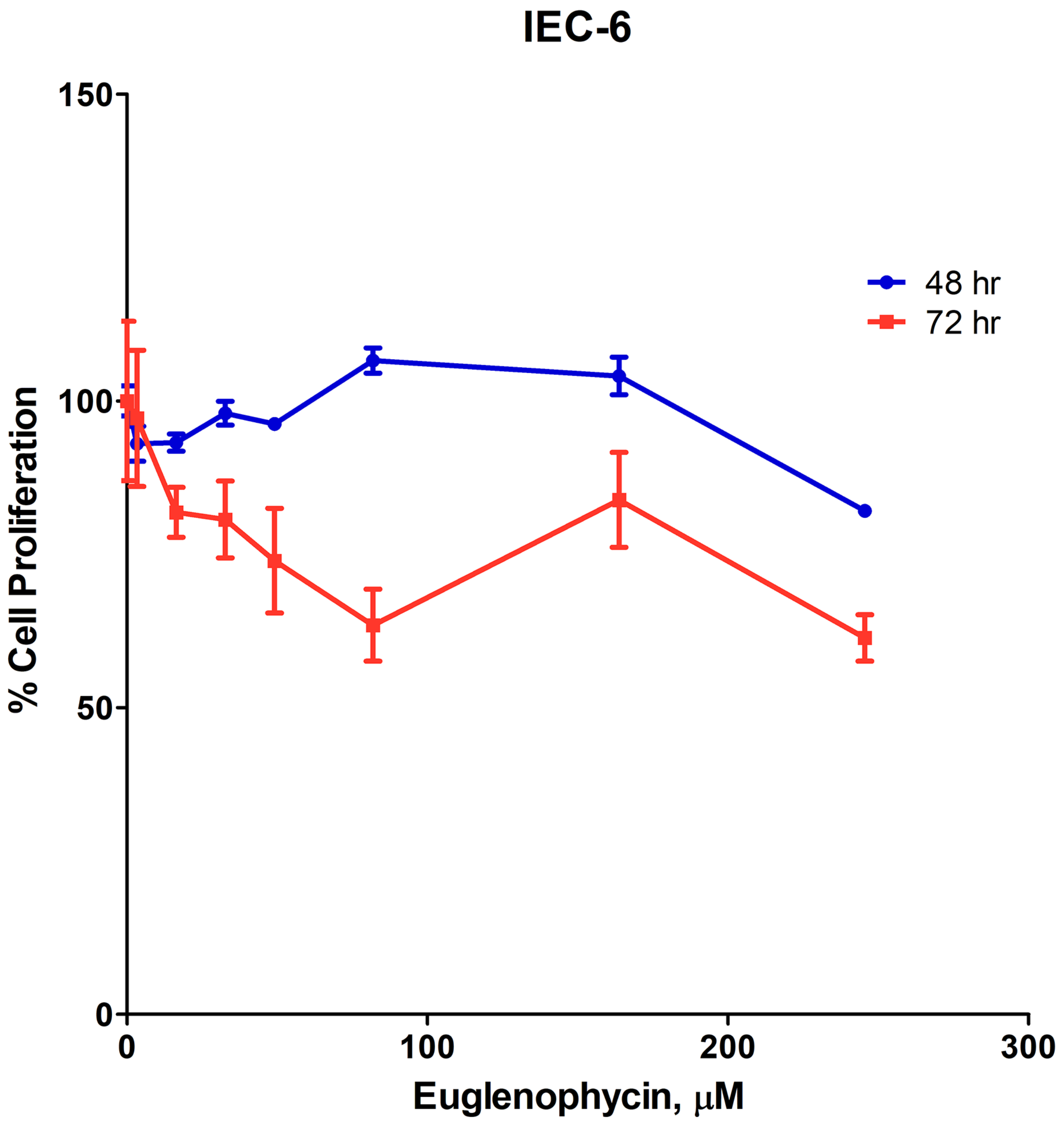

Supplementary Figure 9: Effects of euglenophycin on the proliferation of IEC-6 "normal" rat intestinal epithelial cells.

Supplementary Table 1: Primers used for qRT-PCR targeting autophagy markers and *Gapdh*

Target gene	Primer Code	Sequence (5' – 3')
Gapdh	FH2_GAPDH	cttttgcgtcgccag
	RH2_GAPDH	ttgatggcaacaatatccac
Lc3a	FH1_MAP1LC3A	agaaaggattttgaggaggg
	RH1_MAP1LC3A	ttcatctgcaaaactgagac
Lc3b	FH1_MAP1LC3B	atagaacgatacaagggtgag
	RH1_MAP1LC3B	ctgtaagcgccttctaattatc
Becn1	FH1_BECN1	cagtatcagagagaatacagtg
	RH1_BECN1	tggaaggttgcattaaagac
Atg5	FH3_ATG5	tgtatgaaagaagctgatgc
	RH3_ATG5	tgtcattttgcaatcccatc
Atg12	FH1_ATG12	ctctctatgagtgttttggc
	RH1_ATG12	cacatctgttaagtctcttgc
